# Supplementary material for: Cosmetic Application of Cyanobacteria Extracts with a Sustainable Vision to Skincare: Role in the Antioxidant and Antiaging Process
Source: Mar Drugs. 2022 Dec 2;20(12):761. doi: 10.3390/md20120761 (PMC9785593; doi:10.3390/md20120761)

Figure S1: Keratinocyte (HaCat) viability after 24 and 48 h of incubation with cyanobacteria aqueous (a-g) and acetone (h-n) extracts. (a, h) *Cyanobium* sp. LEGE 07175, (b, i) Synechococcales cyanobacterium LEGE 181150, (c, j) *Leptothoe* sp. LEGE 181156, (d, k) Synechococcales cyanobacterium LEGE 181157, (e, l) Synechococcales cyanobacterium LEGE 181158, (f, m) Oscillatoriales cyanobacterium LEGE 181159 and (g, n) *Leptothoe* sp. LEGE 181155. Results are expressed as % of MTT reduction *vs.* the untreated control. DMSO (20%) represents the positive control. Results are expressed as mean ± SD of at least three independent assays, performed in quadruplicate. Statistical differences at **p*<0.05, ***p*<0.01, ****p*<0.001, *****p*<0.0001 (One way ANOVA, Tuckey HSD multiple comparisons test).

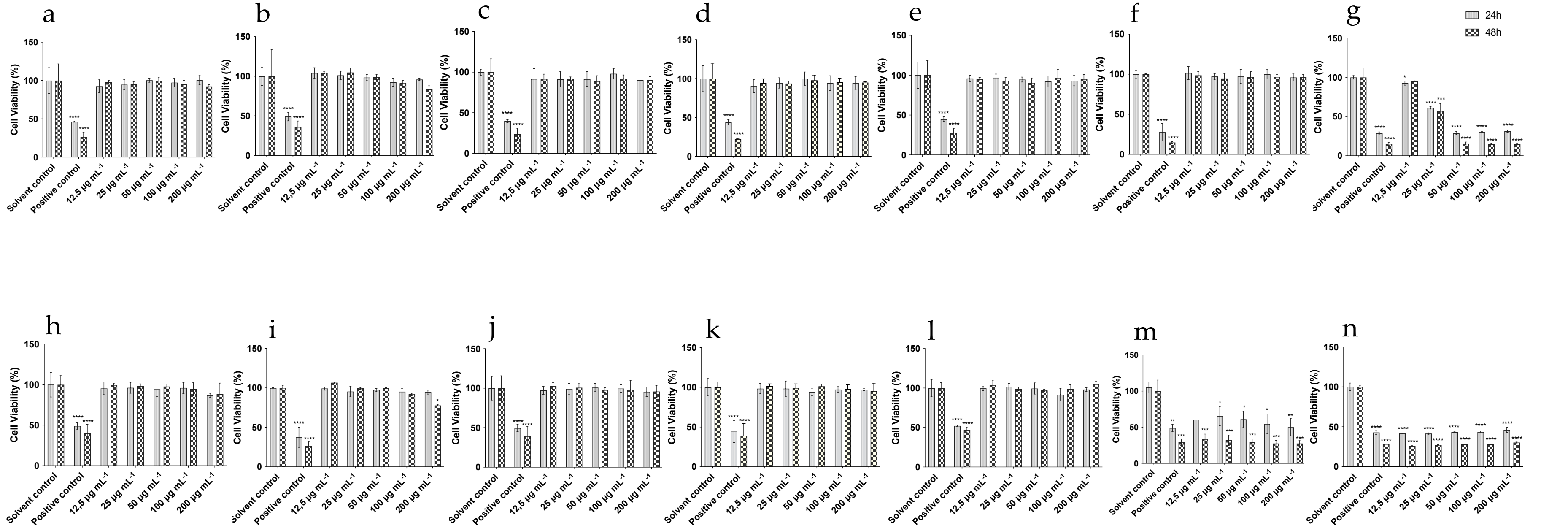

Figure S2: Fibroblast (3T3L1) viability after 24 and 48 h of incubation with cyanobacteria aqueous (a-g) and acetone (h-n) extracts. (a, h) *Cyanobium* sp. LEGE 07175, (b, i) Synechococcales cyanobacterium LEGE 181150, (c, j) *Leptothoe* sp. LEGE 181156, (d, k) Synechococcales cyanobacterium LEGE 181157, (e, l) Synechococcales cyanobacterium LEGE 181158, (f, m) Oscillatoriales cyanobacterium LEGE 181159 and (g, n) *Leptothoe* sp. LEGE 181155. Results are expressed as % of MTT reduction vs. the untreated control. DMSO (20%) represents the positive control. Results are expressed as mean ± SD of at least three independent assays, performed in quadruplicate. Statistical differences at *p<0.05, **p<0.01, ***p<0.001, ****p<0.0001 (One way ANOVA, Tuckey HSD multiple comparisons test).

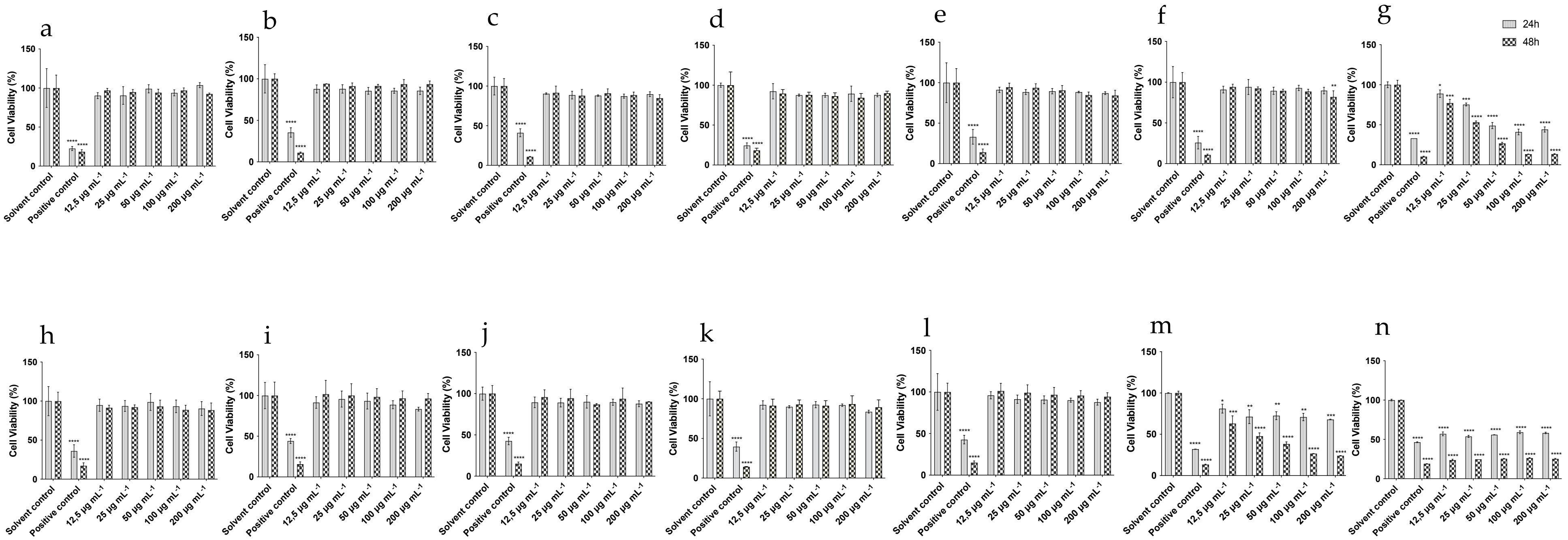

Figure S3: Endothelial cell (hCMEC/d3) viability after 24 and 48 h of incubation with cyanobacteria aqueous (a-g) and acetone (h-n) extracts. (a, h) *Cyanobium* sp. LEGE 07175, (b, i) Synechococcales cyanobacterium LEGE 181150, (c, j) *Leptothoe* sp. LEGE 181156, (d, k) Synechococcales cyanobacterium LEGE 181157, (e, l) Synechococcales cyanobacterium LEGE 181158, (f, m) Oscillatoriales cyanobacterium LEGE 181159 and (g, n) *Leptothoe* sp. LEGE 181155. Results are expressed as % of MTT reduction vs. the untreated control. DMSO (20%) represents the positive control. Results are expressed as mean ± SD of at least three independent assays, performed in quadruplicate. Statistical differences at *p<0.05, **p<0.01, ***p<0.001, ****p<0.0001 (One way ANOVA, Tuckey HSD multiple comparisons test).

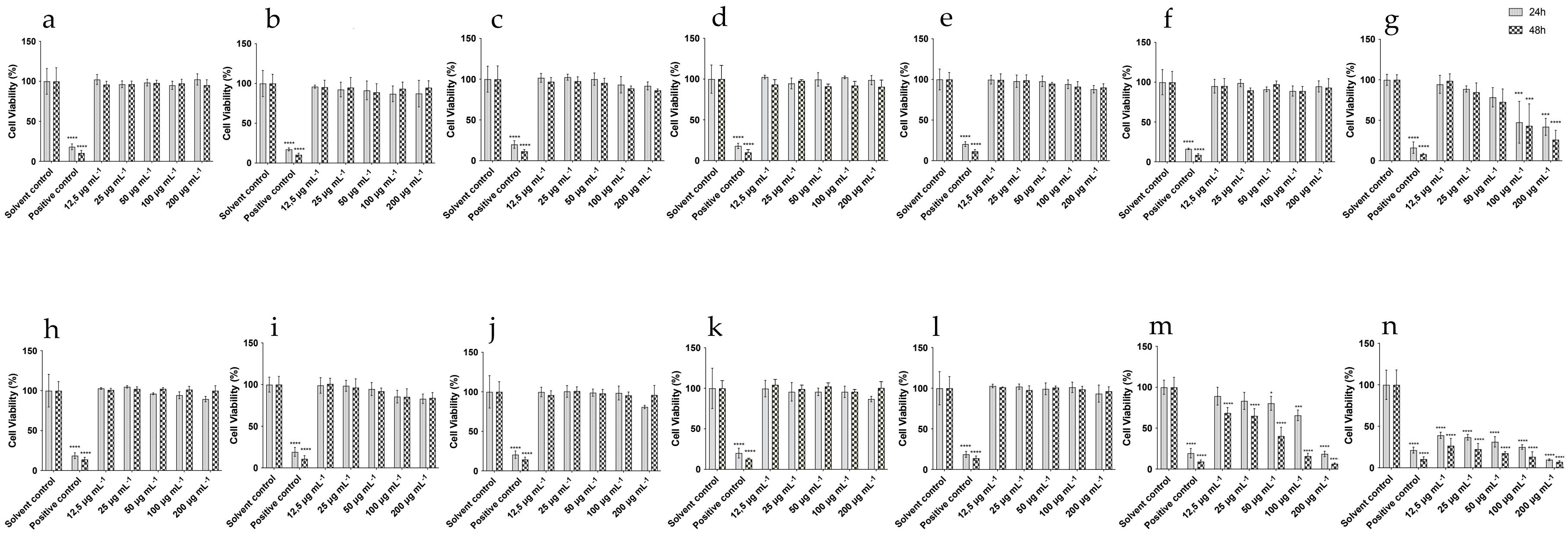

Supplement: Supplementary file 1 [file marinedrugs-20-00761-s001.zip › marinedrugs-2049557-supplementary.pdf]
